# Identification of hub genes for early detection of bone metastasis in breast cancer

**DOI:** 10.3389/fendo.2022.1018639

**Published:** 2022-09-29

**Authors:** Zitong Zhao, Haoran Yang, Guangling Ji, Shanshan Su, Yuqi Fan, Minghao Wang, Shengli Gu

**Affiliations:** Department of Geriatrics, Zhabei Central Hospital, Jing'an District, Shanghai, China

**Keywords:** bioinformatics analysis, breast cancer with bone metastasis, Hub genes, bone microenvironment, bone homeostasis imbalance, immunostaining

## Abstract

**Background:**

Globally, among all women, the most frequently detected and diagnosed and the most lethal type of cancer is breast cancer (BC). In particular, bone is one of the most frequent distant metastases 24in breast cancer patients and bone metastasis arises in approximately 80% of advanced patients. Thus, we need to identify and validate early detection markers that can differentiate metastasis from non-metastasis breast cancers.

**Methods:**

GSE55715, GSE103357, and GSE146661 gene expression profiling data were downloaded from the GEO database. There was 14 breast cancer with bone metastasis samples and 8 breast cancer tissue samples. GEO2R was used to screen for differentially expressed genes (DEGs). The volcano plots, Venn diagrams, and annular heatmap were generated by using the ggplot2 package. By using the cluster Profiler R package, KEGG and GO enrichment analyses of DEGs were conducted. Through PPI network construction using the STRING database, key hub genes were identified by cytoHubba. Finally, K-M survival and ROC curves were generated to validate hub gene expression.

**Results:**

By GO enrichment analysis, 143 DEGs were enriched in the following GO terms: extracellular structure organization, extracellular matrix organization, leukocyte migration class II protein complex, collagen tridermic protein complex, extracellular matrix structural constituent, growth factor binding, and platelet-derived growth factor binding. In the KEGG pathway enrichment analysis, DEGs were enriched in Staphylococcus aureus infection, Complement and coagulation cascades, and Asthma. By PPI network analysis, we selected the top 10 genes, including SLCO2B1, STAB1, SERPING1, HLA-DOA, AIF1, GIMAP4, C1orf162, HLA-DMB, ADAP2, and HAVCR2. By using TCGA and THPA databases, we validated 2 genes, SERPING1 and GIMAP4, that were related to the early detection of bone metastasis in BC.

**Conclusions:**

2 abnormally expressed hub genes could play a pivotal role in the breast cancer with bone metastasis by affecting bone homeostasis imbalance in the bone microenvironment.

## Introduction

Globally, among all women, the most frequently detected and diagnosed and the most lethal type of cancer is breast cancer (BC) (1). Distant metastasis and drug resistance represent the major causes of death in patients with BC (2–6). In particular, bone is one of the most frequent distant metastases in BC patients and bone metastasis arises in approximately 80% of advanced BC patients (7). Bone metastasis often occurs concomitantly with severe pain, pathologic bone fracture, nerve compression, and hypercalcemia leading to a reduced quality of life. According to ENVISION consensus statement (8), the Risk-stratified early detection of bone metastasis in patients with BC is helpful for the treatment. We need to screen and validate early markers for bone metastasis that can distinguish metastasis from non-metastasis BC.

To achieve this primary objective, numerous wet and dry experimental approaches have been developed. Previous literature suggests that, both *in vitro* and *in vivo*, PPARy coactivator-1a(PGC-1c) regulates cell migration and invasion of breast cancer (9). Hoxa5microrna-26a-5p promotes osteosarcoma cell proliferation and migration by targeting *in vitro* and *in vivo* (10). Previous literature suggests that, both *in vitro* and *in vivo*, paeoniflorin regulates the migration and invasion of breast cancer cells by down-regulating microrNA-15B through FOXO1/CCND1/β-catenin axis (6). Previous studies have also indicated that CEP55 was a key regulator in BC spinal metastases by Bioinformatics Analyses (11). Despite the significant developments and rapid progression in the field of BC with bone metastasis, translation of this information into early detection markers and effective treatments are limited.

In recent, accompanied by the increasing advancements made in the fields of bioinformatics tools and high-throughput sequencing technologies (12), our predictions of tumorigenesis, development, and distant metastases of various types of tumors can become more accurate.

Specifically speaking, the widely use of high-throughput platforms applies to early diagnosis, prediction screening, prognosis, and individualized prevention and treatment (10, 13–18). Nevertheless, multiple factors, including heterogeneity across samples, and different screening methodologies. statistical models, mining techniques, and the effect caused by small sample sizes in independent studies may lead to false-positive (FP) or false-negative (FN) findings. In order to overcome these limitations, it has been believed that integrated bioinformatics analysis based on collective datasets is a relatively promising alternative approach. As a result, a considerable number of recent researches have used public datasets successfully. with the help of these datasets such as The Cancer Genome Atlas(TCGA), and Gene Expression Omnibus(GEO), these studies identified diagnostic markers, prognostic markers, and markers to monitor response to cancer therapies (19–24). For example, Qiang R, et al. found that there are five hub genes(CDK1, CDC20, CCNB1, CENPF, and MAD2L1) that have a close relation to the occurrence and development of Hepatitis B virus-associated hepatocellular carcinoma through the mining of a public microarray dataset GSE55092 and GSE121248 (25).

Hence, Multi-datasets combined analysis and database mining have become an important part of molecular biology research. In recent days, Numerous investigations have tried to explain the mechanism of bone metastasis in BC by Bioinformatics analysis (26–28). However, these researches have some defects and problems, for example, the sample size of the dataset is small, most of them are single-source datasets, and the latest data have not been fully studied yet. As a result, in order to provide a new basis for predicting and diagonizing bone metastasis in BC, datasets must be integrated and analyzed as a whole.

To achieve this goal, the first step we did was to explore and analyze the key differentially expressed genes (DEGs) associated with BC with bone metastasis. We performed an integrated bioinformatics analysis based on the GEO and TCGA datasets. Next, we performed functional and pathway enrichment analyses of DEGs, which were also integrated to construct a protein-protein interaction (PPI) network and screen hub genes.

Afterward, we have a tendency to evaluate the prognostic value of the hub genes in TCGA. At the end of this research, key hub genes were identified, and we used imaging evidence of immunohistochemistry in the Human Protein Atlas database (29) (THPA. https://www.proteinatlas.org/) to validate key hub genes. Accordingly, there are 2 main aims in this paper. The first objective was to explain and explore the mechanism of bone metastasis of BC. Our second goal was to identify the key gene to predict breast cancer metastasis to bone. So, we did this research to help the treatment of BC bone metastasis. The flow chart of the present experiment was shown in **Supplementary Figure 1**.

## Material and methods

### Microarray dataset selection

The present study selected GSE55715, (30) GSE103357 (30), and GSE146661 (31) gene expression profiling data, which were never systemically studied, from the GEO database (https://www.ncbi.nlm.nih.gov/geo/). Essential information of the included datasets was shown in **Table 1**. This study wasn’t conducted on human tissue specimens, and 3 sets of microarray data were downloaded from GEO. Due to this, consistent with any current decree in China, the analysis didn’t need associate Institutional ethical review board or Human Research Ethics Committee approval or patient consent.

**Table 1:** Basic information of the microarray datasets.

ID	Platform	Data type	Author	update date	Country	Sample type	n(BM)	n(primary)
GSE55715	GPL6947	mRNA	Johnson, Bessette et	Feb 01, 2021	Australia	Human tissues	3	2
GSE103357	GPL6947	mRNA	Johnson, Bessette et	Jul 25, 2021	Australia	Human tissues	3	2
GSE146661	GPL11532	mRNA	Montaudon, Nikitorowicz-Buniak et	Aug 31, 2020	France	Human tissues	8	4

BM, bone metastasis; ID, identification.

### Identification of DEGs

GEO2R (http://www.ncbi.nlm.nih.gov/geo/geo2r) is a Web-based (WEB) interactive tool with R and was used to identify DEGs between BC with bone metastasis and BC but no metastasis. Based on the statistical significance threshold of adj. p-value < 0.05 and |log_2_ fold‐change| > 1, the significant DEGs between groups were screened. Volcano plots were made by using R statistical software (R Core Team, version 3.6.3). and packages ggplot2 (32) in R and Venn diagrams were generated by using the same tool. Next, to help us better understand DEGs, correlation analysis was applied and conducted by using Spearman correlation analysis and visualized by adopting the ggplot2 package. Lastly, the ggplot2 package was employed to create the annular heatmap.

### Gene ontology and Kyoto encyclopedia of genes and genomes pathway enrichment analysis of DEGs

GO analysis comprises a biological process (BP), cellular component (CC), and molecular function (MF). The GO enrichment analysis of DEGs in bone metastasis samples of BC was performed by the cluster profile (33) R package. The KEGG pathway enrichment analysis of DEGs was performed using the same R package (33). Afterward, we used the org.Hs.eg.db (version 3.4.0) and GOplot R(version 1.0.2) packages for analysis and visualization of the results by generating cluster plots (34),

### Construction of the predicted PPI network

The PPI networks consist of protein complexes formed by biochemical events and/or electrostatic forces, which have unique biological functions as complexes. The PPI network of an organism is the skeleton of its signaling circuits that mediate cellular responses to environmental and genetic cues. Understanding this circuitry could improve the prediction of cellular behavior and gene function in response to different signals. The Search Tool for the Retrieval of Interacting Genes(STRING; https://string-db.org/), a well-known online biological tool for the prediction of PPI, comprises direct (physical) and indirect(functional) associations (35). With the help of version 11.0 of the PPI database STRING, we identified the DEGs involved in the PPI. In this PPI network, the required interaction score for determining a significant interplay was medium confidence (0.400) as the cut-off criteria. Second, the PPI network was visualization with Cytoscape software (36)(version 3.8.2). Finally, the plug-in cytohubba was used to find out the hub genes among the screened DEGs. The CytoHubba (37) is a plug-in for the Cytoscape program, whose main function was to screen out genes with bone metastasis risk of the PPI network using the Clustering Coefficient method.

### Validation of Hub gene expression

We analyzed the TCGA dataset to validate and verify the potential prognostic role of the ten hub genes in bone metastasis of BC. The TCGA dataset, together with other clinically relevant information from 1222 samples (38), furnished the RNA-Seq (level 3, HTSeq-FPKM) data, including tumor growth and metastasis. We investigated the interrelation between the expression level of hub genes and tumors. With the help of the Wilcoxon rank-sum test, we analyzed the difference in the expression of hub genes between BC samples and adjacent normal tissues. Based on patients’ expression level and median value of the hub gene, patients with BC were clustered into 2 groups, the high or low expression group. The results were generated with violin plots. And using the ggplot2 R package, boxplots were plotted.

### Survival analysis to screen the hub genes

In short, using the R packages survival and survminer to plot Kaplan-Meier (K-M) survival curves, survival analysis was carried out. It was The K-M survival curves that were used to represent the progression-free interval (PFI)distributions between BC patients with bone and no metastasis. Using the PFl time obtained from TCGA, the relations of gene expression with patients’ survival outcome were calculated. Following that, in order to further appraise the results of the K-M survival analysis, receiver operating characteristic (ROC) curves were performed using the pROC package in R language (39).

### Immunohistochemistry-based validation of hub genes in THPA

THPA, a public database that includes over 5 million immunohistochemically stained tissues and cells distribution information for 26,000 human proteins, was a program supported by a grant from Sweden. THPA can examine normal and BC tissues by antibody proteomics can be examined by THPA, which is often used for the validation of the hub target genes’ expressions (24). Therefore, we used this pathology tool to evaluate expression levels of hub genes between normal tissues and BC tissues from THPA.

## Results

### Identification of DEGs in BC

**Figure 1:**
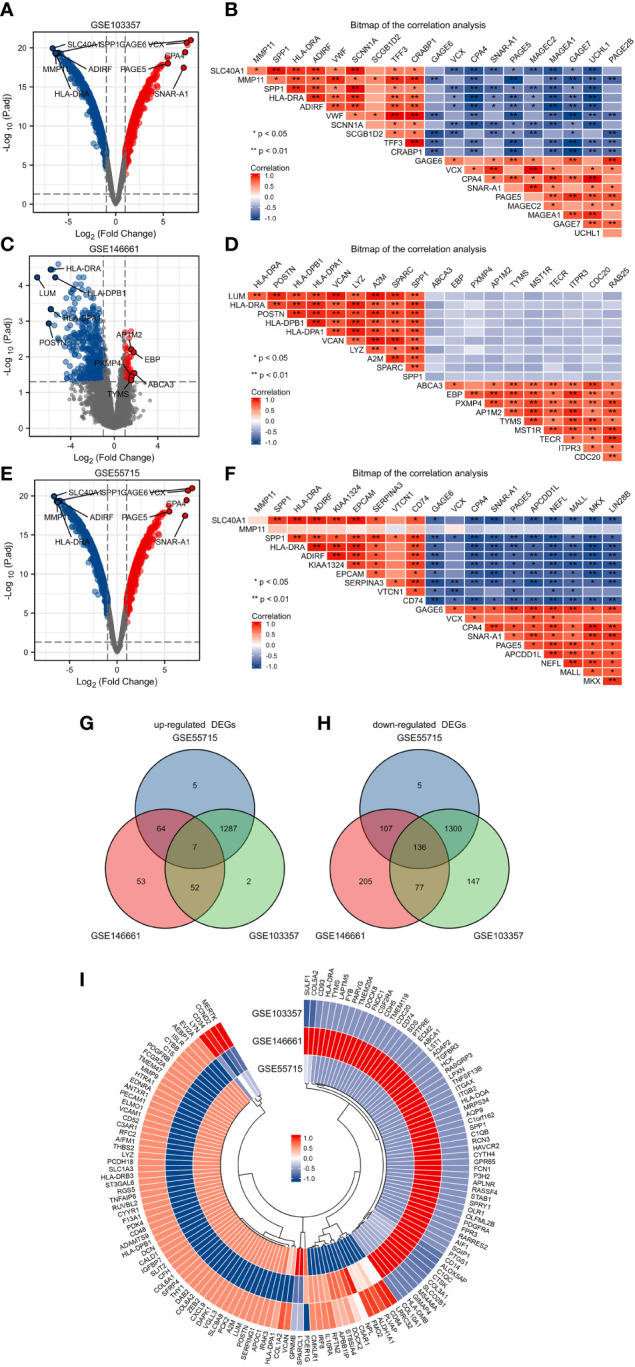
Identification of DEGs in GSE103357, GSE146661 and GSE55715. **(A)** The volcano plots showing all the expressed genes from GSE103357. **(B)** The heatmap of the correlation analysis between the top 10 up or downregulated DEGs in GSE103357. **(C)** The volcano plots showing all the expressed genes from GSE146661. **(D)** The heatmap of the correlation analysis between the top 10 up or downregulated DEGs in GSE146661. **(E)** The volcano plots showing all the expressed genes from GSE55715. **(F)** The heatmap of the correlation analysis between the top 10 up or downregulated DEGs in GSE55715. **(G, H)** The Venn diagram of up-regulated and down-regulated DEGs. **(I)** The annular heatmap of 143 overlapping DEGs. Blue represents downregulated genes, and red represents upregulated genes in the volcano plots. Red and blue denoted positive and negative correlation in the Correlation heatmap. Each column represents one dataset, and each row represents one gene in the annular heatmap. Blue represents downregulated genes and red represents upregulated genes in the annular heatmap. DEGs: differentially expressed gene.

We compared microarray gene expression datasets GSE55715, GSE103357, and GSE146661, which were obtained from the GEO database, between patients with primary BC and BC patients with bone metastatic. Based on the identification of the microarray results of the GSE55715 datasets, 1403 up-regulated and 1548 down-regulated genes were identified in GSE55715. 3048 DEGs were identified in the GSE103357 dataset (1388 upregulated,1660 downregulated), while 704 were identified in the GSE146661 dataset (176 upregulated, 528 downregulated). The volcano plot of the dataset was delineated for the visualization of DEGs in **Figures 1A, C, E**, and the top 10 up-or down-regulated DEGs were selected for Spearman correlation analysis. **Figures lB, D, F** show a heatmap of the correlation analysis between the DEGs. In total, 7 up-regulated DEGs and 136 down-regulated DEGs were shared the three datasets, as identified through Venn diagram analyses in **Figures 1G, H**. The differentially expressed genes was shown in **Figure 1I** by the heatmap. The detailed results of DEGs are shown in **Table S1**

### KEGG and GO enrichment analysis of DEGs

**Figure 2:**
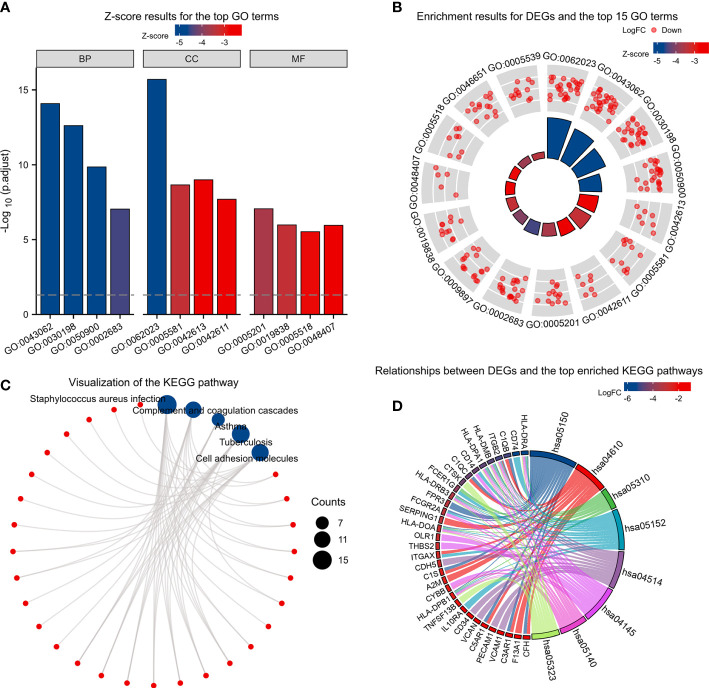
KEGG and GO enrichment analysis of DEGs. **(A)** Z-score results for the top 12 GO terms, including the top 4 BPs, CCs, and MFs. **(B)** Enrichment results for DEGs and the top 15 GO terms. **(C, D)** Relationships between DEGs and the top enriched KEGG pathways by different methods. Z-scores were defined as follows:(upregulated genes–downregulated genes)/total genes.DEGs: differentially expressed genes; GO: Gene Ontology; BP: biological process; CC: cellular component; MF: molecular function. KEGG: Kyoto Encyclopedia of Genes and Genomes.

Based on the GO enrichment analysis,143 overlapping DEGs were enriched for 513 BP terms,63 CC terms, and 43 MF terms. Under BP terms (**Figure 2A**), DEGs were mainly enriched according to the following processes: extracellular structure organization, extracellular matrix organization, and leukocyte migration. For CC terms(**Figure 2A**), DEGs were primarily enriched in the collagen-containing extracellular matrix, major histocompatibility complex (MHC) class II protein complex, and collagen trimer. It was revealed from Enrichment analysis of MF terms(**Figure 2A**) that most DEGs were enriched in extracellular matrix structural constituent, growth factor binding, and platelet-derived growth factor binding. The relationship between DEGs and GO terms were exhibited in **Figure 2B** and **Table 2**. The enrichment analysis of KEGG pathways (**Figures 2C, D**, **Table 3**) included 32 KEGG pathways, and most of the DEGs were enriched significantly in staphylococcus aureus infection, complement and coaqulation cascades, asthma, tuberculosis, and cell adhesion molecules.

**Table 2:** GO enrichment analyses table.

Ontology	ID	Description	GeneRatio	BgRatio	pvalue	p.adjust	qvalue
BP	GO:0043062	extracellular structure organization	27/135	422/18670	3.18399E-18	8.32296E-15	6.51881E-15
BP	GO:0030198	extracellular matrix organization	24/135	368/18670	1.88302E-16	2.46111E-13	1.92762E-13
BP	GO:0050900	leukocyte migration	24/135	499/18670	1.60713E-13	1.40035E-10	1.0968E-10
CC	GO:0042613	MHC class II protein complex	7/140	16/19717	8.48361E-12	1.02652E-09	7.6799E-10
CC	GO:0005581	collagen trimer	11/140	87/19717	2.74712E-11	2.21601E-09	1.65791E-09
CC	GO:0042611	MHC protein complex	7/140	25/19717	3.37985E-10	2.04481E-08	1.52983E-08
MF	GO:0005201	extracellular matrix structural constituent	13/131	163/17697	2.40906E-10	8.79308E-08	7.75972E-08
MF	GO:0019838	growth factor binding	11/131	137/17697	5.74465E-09	1.0484E-06	9.25191E-07
MF	GO:0048407	platelet-derived growth factor binding	5/131	11/17697	9.177E-09	1.11654E-06	9.85321E-07

**Table 3:** KEGG enrichment analyses table.

Ontology	ID	Description	GeneRatio	BgRatio	pvalue	p.adjust	qvalue
KEGG	hsa05150	Staphylococcus aureus infection	15/87	96/8076	5.68e-14	8.75e-12	7.00e-12
KEGG	hsa04610	Complement and coagulation cascades	11/87	85/8076	1.32e-09	1.02e-07	8.15e-08
KEGG	hsa05310	Asthma	7/87	31/8076	2.81e-08	1.44e-06	1.15e-06
KEGG	hsa05152	Tuberculosis	13/87	180/8076	5.27e-08	1.66e-06	1.33e-06
KEGG	hsa04514	Cell adhesion molecules	12/87	149/8076	5.39e-08	1.66e-06	1.33e-06

### PPI network construction and analysis of hub genes

**Figure 3:**
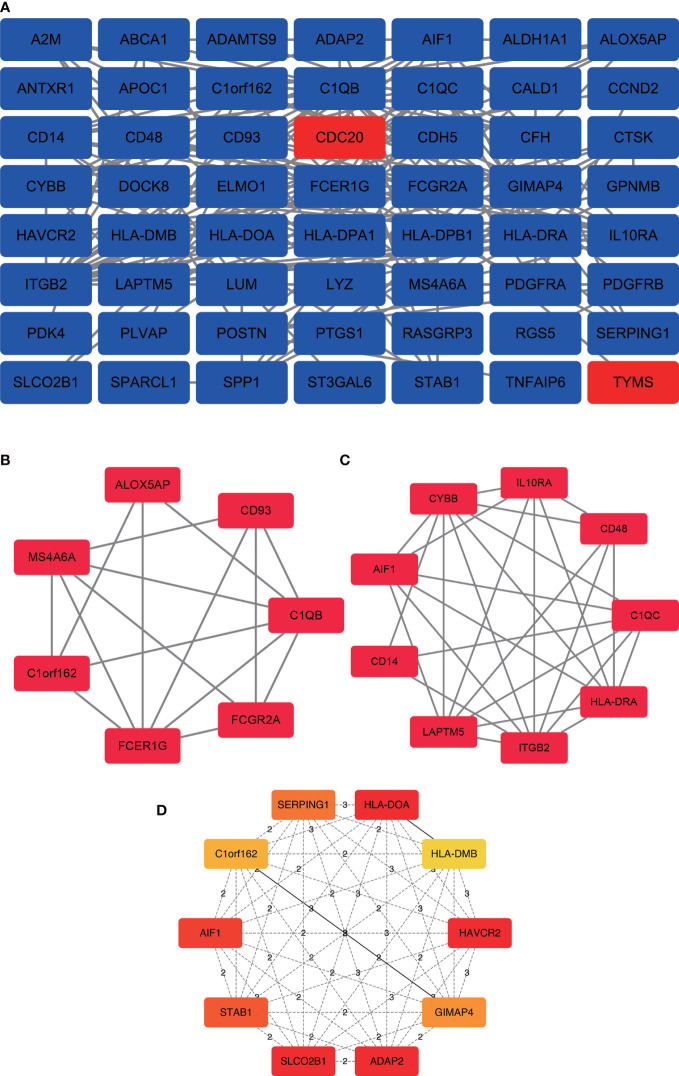
PPI network construction and analysis of hub genes. **(A)** The most significant module was obtained from the PPI network with 56 nodes and 178 edges. **(B)** Module 1 contained 7 upregulated genes nodes and 16 edges, MCODE score=5.33. **(C)** Module 2 contained 9 gene nodes and 28 edges, MCODE score=7. **(D)** The hub genes were selected from the PPI network using the cytoHubba plugin. DEGs, differentially expressed genes; PPI, protein–protein interaction.

In the PPI network, a total of 143 DEGs were included, which originated from the STRING database. The construction of PPI network aimed to further understand the interactions of DEGs correlated with bone metastasis risk, consisting of 56 nodes and 178 edges (**Figure 3A**). Furthermore, from the PPI network, the 2 significant modules(score>4.5) were extracted. Module 1 contained 7 upregulated genes nodes and 16 edges (**Figure 3B**). Module 2 contained 9 gene nodes and 28 edges (**Figure 3C**). The cytoHubba plugin selected the top 10 genes(**Figure 3D**) ranked by the Clustering Coefficient method as hub genes, including SLCO2B1, STAB1, SERPING1, HLA-DOA, AIF1, GIMAP4, C1orf162, HLA-DMB, ADAP2, and HAVCR2.

### Hub gene expression of BC patients

**Figure 4:**
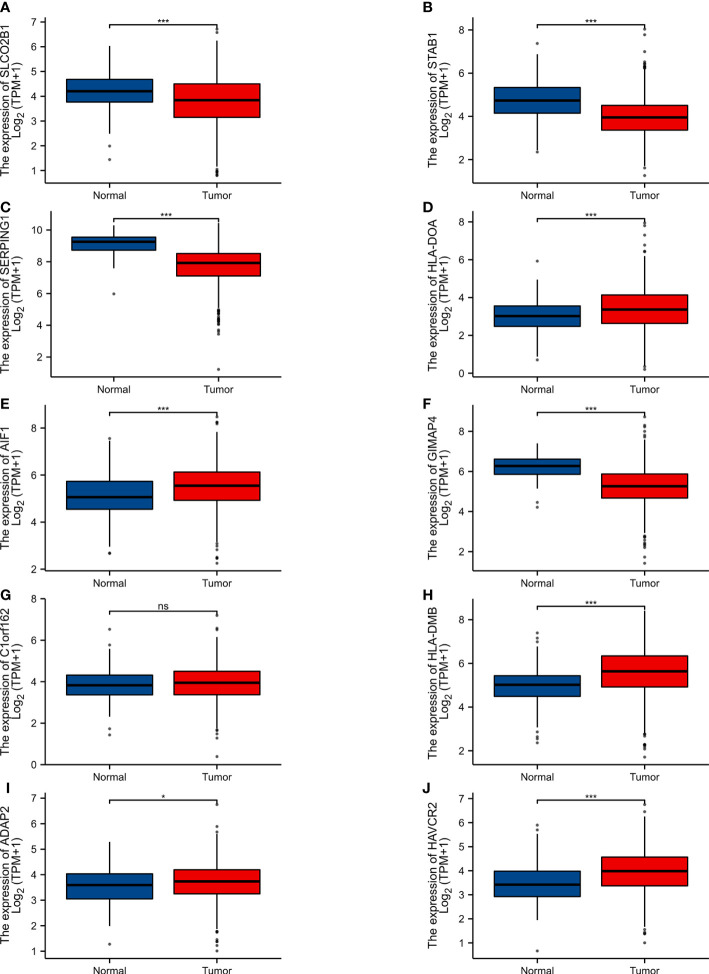
Expression of various genes in the normal samples and tumour samples. “Normal” represents the normal tissue and “Tumour” represents the tumour samples **(A)** SLCO2B1 (P < 0.001). **(B)** STAB1 (P < 0.001). **(C)** SERPING1 (P < 0.001). **(D)** HLA-DOA (P < 0.001). **(E)** AIF1 (P < 0.001). **(F)** GIMAP4 (P < 0.001). **(G)** C1orf162 (P > 0.05). **(H)** HLA-DMB (P < 0.001). **(I)** ADAP2 (P = 0.027). **(J)** HAVCR2 (P < 0.001). ns, p≥0.05; *, p< 0.05; ***, p<0.001.

The expression level of 10 hub genes was confirmed by using the TCGA dataset. Among them,5 genes (HLA-DOA, AIF1, HLA-DMB, ADAP2, AVCR2) were upregulated, and 4 genes (SLCO2B1, STAB1, SERPING1, GIMAP4) were downregulated. In addition, the differences of C1orf162 expression are not statistically significant (**Figure 4**).

### Survival analysis of selected hub genes

**Figure 5:**
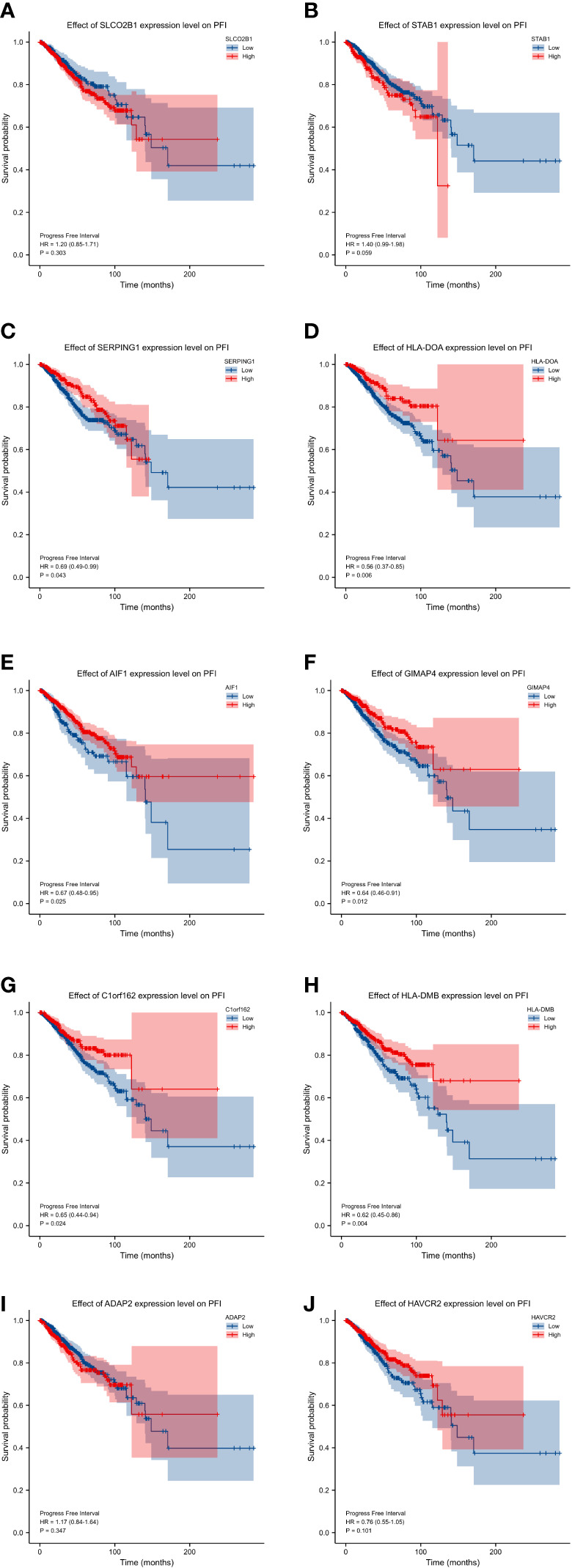
progression-free interval (PFI) curves of the 10 hub genes. progression-free interval (PFI) curves by high and low expression of various genes in BC patients. **(A)** SLCO2B1 **(B)** STAB1 **(C)** SERPING1 **(D)** HLA-DOA **(E)** AIF1 **(F)** GIMAP4 **(G)** C1orf162 **(H)** HLA-DMB. **(I)** ADAP2 **(J)** HAVCR2.

**Figure 6:**
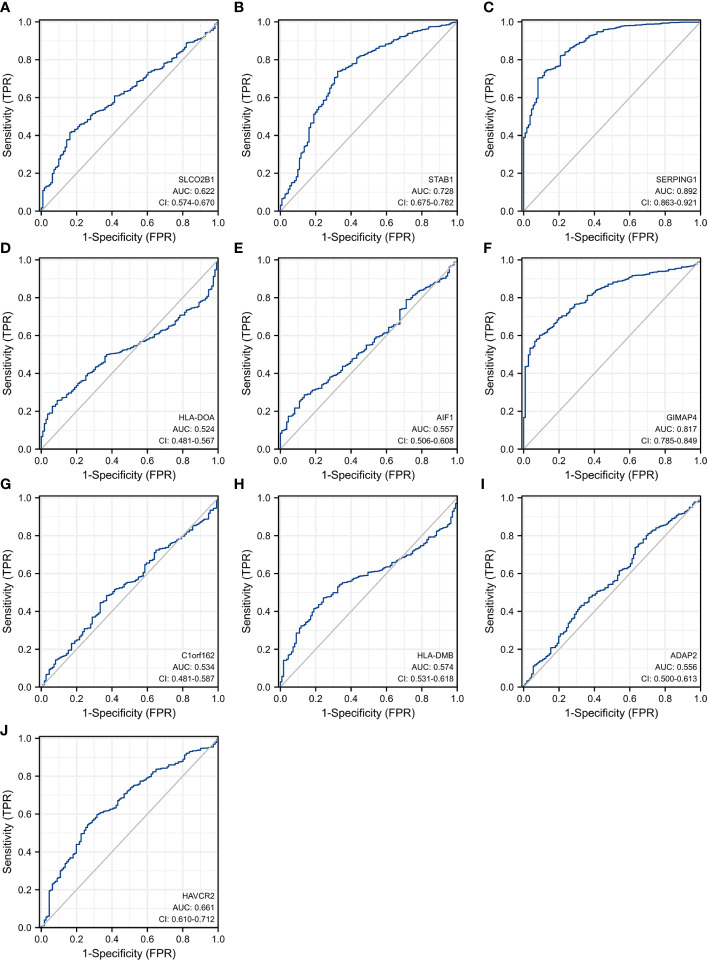
Receiver operating characteristic curves for the 10 hub genes. **(A)** SLCO2B1 (AUC = 0.622, CI = 0.574–0.670) **(B)** STAB1 (AUC = 0.728, CI = 0.675–0.782) **(C)** SERPING1 (AUC = 0.892, CI = 0.863–0.921) **(D)** HLA-DOA (AUC = 0.524, CI = 0.481–0.567) **(E)** AIF1 (AUC = 0.557, CI = 0.506–0.608) **(F)** GIMAP4 (AUC = 0.817, CI = 0.785–0.849) **(G)** C1orf162 (AUC = 0.534, CI = 0.481–0.587) **(H)** HLA-DMB (AUC = 0.574, CI = 0.531–0.618) **(I)** ADAP2 (AUC = 0.556, CI = 0.500–0.613) **(J)** HAVCR2 (AUC = 0.661, CI = 0.610–0.712).

**Figure 7:**
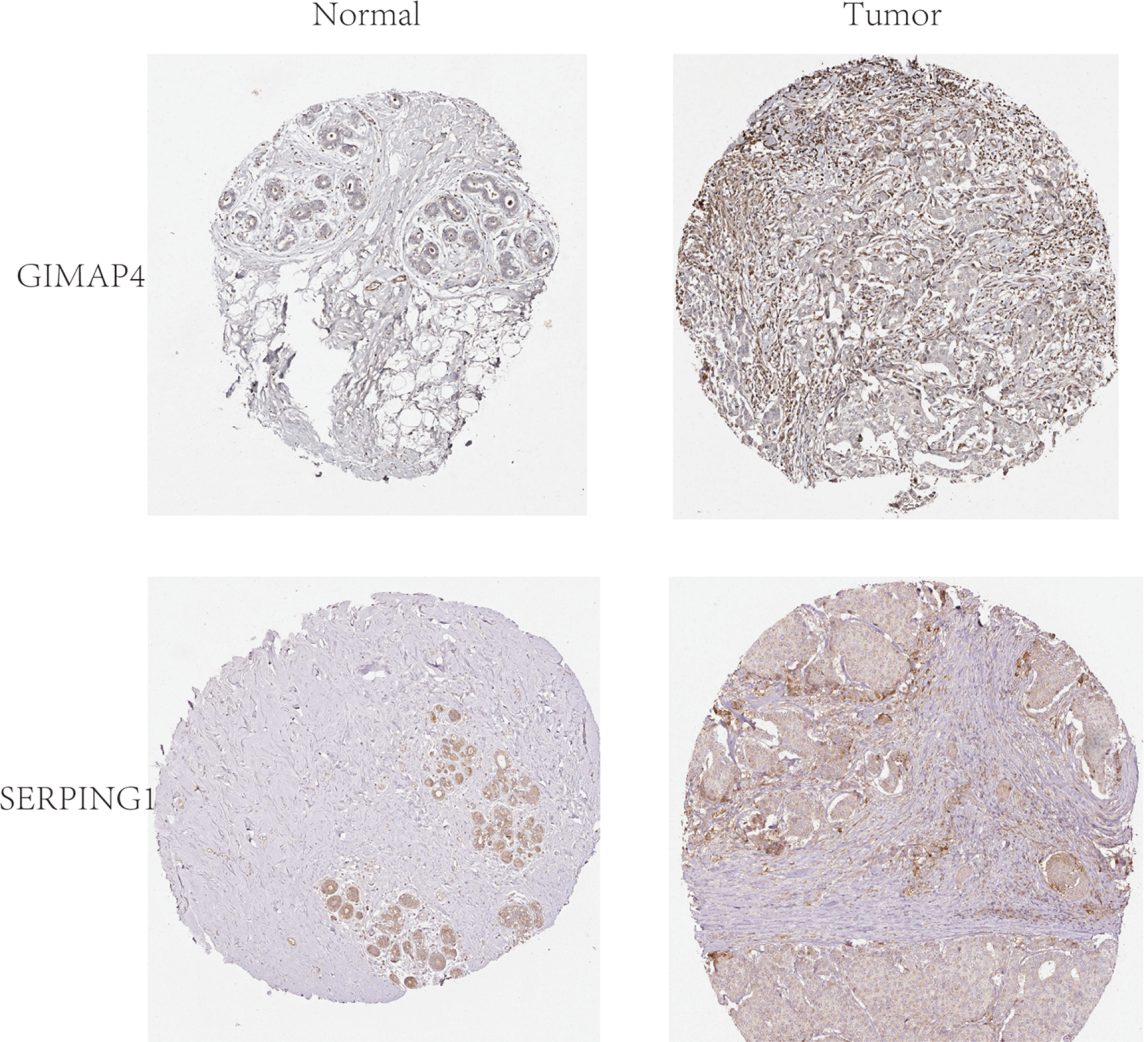
Representative histological images from the Human Protein Atlas database (THPA, https://www.proteinatlas.org/). Normal breast tissue with staining for GIMAP4 was obtained from a female subject aged 23 years (Patient id: 2773; staining: not detected; intensity: negative; quantity: none; location: none; magnification, not available), and BC tissue was obtained from a female patient aged 27 years (Patient id: 2392; staining: not detected; intensity: negative; quantity: none; location: none; magnification, not available).Normal breast tissue with staining for SERPING1 was obtained from a female subject aged 24 years (Patient id:3856; Staining: Medium; Intensity: Moderate; Quantity:>75%; Location: Cytoplasmic/membranous; magnification, not available), and BC tissue was obtained from a female patient aged 75 years (patient ID: 2428; staining: Low; intensity: Weak; quantity: 75%-25%; Location: Cytoplasmic/membranous; magnification, not available).

In order to explore and validate the prognostic value of these hub genes, further research is needed. R language was used to perform a survival analysis of the 10 genes in the 1222 samples from TCGA by using the K-M plotter (**Figure 5**). Based on our K-M survival curve for PFI, patients with low expression of SERPING1, AIF1, HLA-DMB, HLA-DOA and GIMAP4 had shorter PFI times. Because P values are more than 0.05, SLCO2B1, STAB1, ADAP2, and HAVCR2 genes were not statistically significant. Subsequently, we generate and analyze ROC curves by PFI to achieve a complete view of the hub genes’ predictive value. The outcomes revealed that two hub genes (SERPING1 and GIMAP4) were negatively correlated with breast cancer bone metastasis (**Figure 6**). What’s more, representative images indicated that the expression of SERPING1 was downregulated, and further study is needed for the expression of GIMAP4 in BC tissues (**Figure 7**).

## Discussion

Female BC, surpassing lung cancer, has become the most commonly diagnosed malignancy, with an estimated 2.3 million new cases(11.7%) and an estimated 0.7 million deaths(6.9%) (40). Despite improvements in the diagnosis and treatment of breast cancer, the prognosis of bone metastasis in BC patients is poor with shorter survival. In order to a better understanding of the underlying mechanisms for BC with bone metastasis, researches have been conducted by professor and scientist around the world. The study of Maroni, P., et al. demonstrated that Interleukin (IL) -6, IL-8, IL-1β, and IL-11 promoted the bone metastasis of BC by promoting the osteolytic vicious cycle of bone resorption and tumor growth (41). Studies on the hemocompatibility and osteocompatibility of electroplated composite coating showed that this technique can be used to repair bone defects caused by metastatic bone tumors (42). The study by Mou H et al. found that high serum alkaline phosphatase and lactate dehydrogenase were associated with bone metastasis and poorer prognosis (43). Despite such findings, the solution to predict the bone metastasis of breast cancer patients is still lacking. Through performing bioinformatics integrated analysis based on the GEO dataset, we aimed to provide new insights into the molecular mechanism and find out new markers to predict metastases for BC bone metastasis.

Indeed, this approach has previously been widely applied to systematically identify metastasis-associated genes. Mou H et al. demonstrated that core driver genes (BNIP3 and the lncRNA RP11-317-J19.1) were related to the bone metastasis of breast cancer by using the bioinformatics approach in the GSE66206 dataset (28). The study by Luo Y et al. suggests that miR-624-5p was a risk factor for tumor metastasis in osteosarcoma progression by the GSE65071 dataset (44). Despite the use of a single dataset, these studies had achieved good results. The study of Wan D. et al. found that overexpression of LINC00691 regulated the function of Mir-1256 through the mechanism of ceRNA, and promoted the expression of ST5 to further affect the development and metastasis of osteosarcoma (17).

In order to overcome the shortcomings caused by the small sample size and heterogeneity of the studied group, several public electronic databases, such as GEO and TCGA, were analyzed through the use of data mining approaches. In this study, we analyzed 3 GEO datasets (GSE55715, GSE103357and GSE144661) by integrated bioinformatics analysis. In the GSE55715 dataset, we examined 3 BC with bone metastasis samples and 2 primary tumors samples. There were 1403 upregulated and 1548 downregulated genes. In the GSE103357 dataset, we examined 3BC with bone metastasis samples and 2 primary tumors samples. There were 1388 upregulated and 1660 downregulated genes. In the GSE146661 dataset, we examined 8 BC with bone metastasis samples and 4 primary tumors samples. On average, 176 genes were up-regulated and 528 genes were down-regulated from this dataset. Next, 7 upregulated DEGs and 136 downregulated DEGs in three datasets had been identified through Venn diagram analyses. The DEGs were subsequently subjected to GO and KEGG pathway enrichment analysis.

In the GO enrichment analysis, BP analysis of DEGs showed that the genes were associated with extracellular structure organization, extracellular matrix organization, and leukocyte migration. For CC terms. The CC GO terms showed that the genes were related to MHC class II protein complex, collagen trimer, and MHC protein complex. The MF GO terms demonstrated that the genes were related to extracellular matrix structural constituent, growth factor binding, and platelet-derived growth factor binding. By analyzing KEGG enrichment analysis, DEGs were involved in staphylococcus aureus infection, complement and coaqulation cascades, asthma, tuberculosis, and cell adhesion molecules. Then, the PPI network among the identified DEGs was successfully constructed by STRING online database and visualized by Cytoscape software. The main hub genes in the most significant module were SLCO2B1, STAB1, SERPING1, HLA-DOA, AIF1, GIMAP4, C1orf162, HLA-DMB, ADAP2, and HAVCR2.

To increase the credibility of our results, we searched the expression levels and measured the prognostic values of DEGs from a resource of TCGA database. The expression of 9 central genes was found to have the highest correlation with BC based on TCGA. Among them,5 genes (HLA-DOA, AIF1, HLA-DMB, ADAP, AVCR2) were upregulated and 4 genes (SLCO2B1, STAB1, SERPING1, GIMAP4) were downregulated. Further, ROC curve analysis and representative image analysis screened out 2 hub genes (SERPING1 and GIMAP4). Because the low expression of 2 hub genes had short PFI, they were associated with disease progression (bone metastases in BC).

SERPING1 encodes a highly glycosylated plasma protein involved in the regulation of the complement cascade as well as immune response. It is synthesized in the liver, and potentially important in the regulation of crucial physiological pathways including complement activation, blood coagulation, tibrinolysis, and the generation of kinins, Diseases associated with SERPING1 include Angioedema, Hereditary, and Complement Component, Partial Deficiency (45). A recent study by Popeda M., et al. suggested that SERPING1 gene expression was reduced in lymph node metastases of luminal breast cancer patients (46). These previously reported studies combined with the results of our study suggest that SERPING1 may play a part in bone metastases.

GIMAP4 encodes a 37534 Da protein belonging to the GTP-binding superfamily. It also belongs to the immuno-associated nucleotide (IAN)subfamily of nucleotide-binding proteins. Related disease associated with GIMAP4 is Behcet Syndrome (47). It has been reported that GIMAP4 plays a part in the execution of programmed cell death in T cells. Therefore, we surmise that it is associated with tumorigenesis (48). The research of Xu found that GIMAP4 as an immune-related biomarker was associated with the remodeling in cervical cancer tumor microenvironment and served as a potential predictor for distant metastasis (49). In parallel, the study by Mégarbané A., et al. demonstrated that it may play a tumor-suppressive role against BC (50). Consequently, GIMAP4 is also an important indicator of bone metastases and poor outcomes of BC.

An analysis of bioinformatics data indicated that SERPING1 and GIMAP4 are likely to regulate cell adhesion molecules including CD2, CD8A, and SELL (51). Adhesion molecules could promote the bone metastasis of cancer cells (52). Survival is shorter for patients with high levels of adhesion molecule expression. No doubt has an inverse relationship with the SERPING1 and GIMAP4. It was hypothesized that inhibiting SERPING1 and GIMAP4 would promote cell adhesion and consequently promote the destruction of local tumors on bones. Previous studies have shown that neural cell adhesion molecule silencing inhibits osteoblast differentiation by inactivating Wnt/β-catenin and PI3K-Akt signaling pathways. CD137 may be involved in p53 and Wnt/β-catenin signaling pathways, thereby regulating the imbalance of bone homeostasis and affecting bone mass (53, 54). We hypothesized that adhesion molecules may be associated with an imbalance in bone homeostasis. SERPING1 and GIMAP4 are induced by BC-secreted factors in bone metastasis, where it induces an endothelial cell-to-osteoblast transition. The osteogenesis process appears to be regulated by the synaptic interaction between neuronal cells and bone-forming cells in bone-associated neurons (55). Moreover, SERPING1 and GIMAP4 are closely related to bone homeostasis in metastatic bone tumors.

In the present research, hub genes were filtered out among three datasets. Subsequently, survival analysis of the 10 hub genes was performed by using the TCGA database. The research methodology adopted here decreased the risk of random errors arose from the usage of a single dataset and improved the reliability and quality of bioinformatic analysis. In spite of everything, there were also certain limitations associated with the present study in the context of potential clinical application. The first is the small sample size of the validated data obtained from the GEO database, which restrict affects the generalization of the results to other BC patients with bone metastases. Second, the limited medical conditions in Chinese hospitals made the 2 hub genes indicated here not be confirmed in pre-clinical and clinical studies. Future researches will gather more samples to investigate 2 hub genes (SERPING1, GIMAP4) by performing further experiments to test the research possibility in a clinical sample size. *In vitro* and *in vivo* trials are also needed to certify the associations and mechanisms of action of the candidate genes.

## Conclusion

To sum up,143 DEGs associated with BC with bone metastases were identified using bioinformatic analysis. DEGs provided novel and important insight into the mechanisms of BC with bone metastases and advanced our knowledge of the possible mechanisms of pathogenesis and prognosis. Based on the downstream analysis, hub genes (SERPING1, GIMAP4) could play a pivotal role in the bone metastases by affecting bone homeostasis imbalance in the bone microenvironment.

## Data availability statement

The original contributions presented in the study are included in the article/**Supplementary Material**. Further inquiries can be directed to the corresponding authors.

## Ethics statement

Written informed consent was obtained from the individual(s) for the publication of any potentially identifiable images or data included in this article.

## Author contributions

ZZ extracted the data, performed the statistical analysis, and drafted the paper. An investigation of literature and data validation was conducted by HY. GJ contributed to the language revision. SS and YF supplemented the literature and participated in the revision of the article. SG and MW both participated in the literature investigation and statistical analysis, as well as reviewing the manuscript. A version of the manuscript published in its final form has been reviewed and approved by all authors.

## Conflict of interest

The authors declare that this research was conducted in the absence of any commercial or financial relationships that could be construed as a potential conflict of interest.

## Publisher’s note

All claims expressed in this article are solely those of the authors and do not necessarily represent those of their affiliated organizations, or those of the publisher, the editors and the reviewers. Any product that may be evaluated in this article, or claim that may be made by its manufacturer, is not guaranteed or endorsed by the publisher.
