# Supplementary material for: Identification of hub genes for early detection of bone metastasis in breast cancer
Source: Front Endocrinol (Lausanne). 2022 Sep 29;13:1018639. doi: 10.3389/fendo.2022.1018639 (PMC9556899; doi:10.3389/fendo.2022.1018639)
Supplement: Supplementary file 1 [file DataSheet_1.docx]

# Table S1: upregulated and downregulated gene

|  | gene |
| --- | --- |
| Up | RFC2, AIFM1, RUVBL2, TYMS, PCK2, CDC20, MRPS34 |
| Down | SPP1,HLA-DRA,CD74,C1QB,LAPTM5,ITGB2,HLA-DMB,COL3A1,HLA-DPA1,CD93,CD14,C1QC,SLCO2B1,CTSK,MS4A6A,SPARCL1,ALOX5AP,SULF1,COL10A1,GIMAP4,GPNMB,FCER1G,COL5A2,RARRES2,ADAP2,AIF1,C1orf162,APOC1,HLA-DRB3,TGFBR3,LYZ,HAVCR2,LUM,ECM2,FPR3,FCGR2A,PDGFRA,SERPING1,ABCA1,OLFML2B,DCN,TNFAIP6,HLA-DOA,OLR1,SPRY1,CYTH4,SFRP4,THBS2,ITGAX,CDH5,STAB1,AEBP1,SGIP1,FYB,LST1,TMEM119,TMEM204,RCN3,C1S,DOCK8,A2M,HCK,CYBB,PDGFRB,HLA-DPB1,ISLR,PARVG,ANTXR1,POSTN,RASGRP3,HTRA1,PTPRE,TNFSF13B,EDNRA,PTGS1,PCDH18,LPXN,MMP9,RASSF4,APLNR,RGS5,DAB2,AQP9,TMEM47,FNDC1,P3H2,ALDH1A1,IL10RA,FMO2,CCND2,ST8SIA4,MERTK,APBB1IP,IRF8,CD34,NPL,DOCK2,LRRC32,CMKLR1,VCAN,COL1A2,CD84,LYN,C5AR1,PLVAP,RFTN2,SDS,PECAM1,CSF2RA,ELMO1,THY1,IRAK3,COL6A2,ZEB2,VCAM1,CXCL9,CD52,PDK4,SLC1A3,DAPK1,FCN1,CALD1,VGLL3,ST3GAL6,SLC9A9,ADAMTS9,C3AR1,IGFBP7,F13A1,CYYR1,GPR65,SLIT2,EVI2A,COL6A1,CFH,CD48 |

# Supplementary figure 1: The overall implementation framework of the article.
